# A Case of Multifocal Adenoid Cystic Carcinoma of the Breast: Using Immunohistochemical Diagnosis to Guide Conservative Management

**DOI:** 10.7759/cureus.91631

**Published:** 2025-09-04

**Authors:** Diana C Correa-Sandoval, Andrea S Torres-De la Garza, Jose L Guzman-Murguia, Diego A Guajardo-Nieto

**Affiliations:** 1 Research, Breast Care Center, Hospital Angeles Valle Oriente, San Pedro Garza Garcia, MEX; 2 Surgery, Universidad de Monterrey, San Pedro Garza Garcia, MEX; 3 Breast Surgery, Breast Care Center, Hospital Angeles Valle Oriente, San Pedro Garza Garcia, MEX

**Keywords:** adenoid cystic carcinoma, breast, breast cancer, diagnostic and therapeutic challenge, women

## Abstract

Adenoid cystic carcinoma (ACC) of the breast is exceptionally rare and can be misdiagnosed as a more aggressive triple-negative subtype. A 56-year-old woman presented with a painless lesion in the right breast. Imaging (Breast Imaging Reporting and Data System (BI-RADS) 5 mammography and ultrasound) identified four multifocal nodules in the upper outer quadrant at the 10 o'clock position, and core needle biopsies confirmed ACC via immunohistochemistry (negative estrogen receptor (ER), progesterone receptor (PR), human epidermal growth factor receptor 2 (HER2)/neu; positive CD117, cytokeratin 7 (CK7), calponin). She underwent bracket‑guided partial mastectomy with clear margins, followed by adjuvant radiotherapy. Histopathology revealed a 2.5 cm Nottingham Grade I ACC (pT2 pN0 M0) and a 6 mm focus of ductal carcinoma in situ. Despite receptor negativity, endocrine therapy was recommended. Precise immunoprofiling differentiates ACC from basal-like cancers, enabling conservative management. Our case highlights a rare multifocal presentation of ACC managed conservatively with a favorable short-term outcome. This case adds to growing evidence suggesting that, with accurate immunohistochemical diagnosis and multidisciplinary care, breast-conserving surgery may be a safe option in select patients with multifocal ACC. Further multicenter studies are needed to establish management guidelines.

## Introduction

Adenoid cystic carcinoma (ACC) of the breast is an extremely rare neoplasm, accounting for fewer than 0.1% of all breast malignancies [1]. It typically presents in middle-aged to older women and is characterized histologically by a biphasic epithelial-myoepithelial proliferation arranged in tubular, cribriform, or solid patterns [1,2]. Although triple-negative for estrogen receptor (ER), progesterone receptor (PR), and human epidermal growth factor receptor 2 (HER2)/neu, ACC differs from other triple-negative breast cancers by exhibiting indolent clinical behavior and low proliferative activity, typically marked by a Ki-67 index less than 5% [2].

Immunohistochemical markers such as CD117, CK7, and calponin are essential for diagnosis and for distinguishing ACC from more aggressive tumors or benign entities like collagenous spherulosis [3]. Its clinical significance lies in its rarity, diagnostic complexity, and therapeutic implications. Given its rarity, ACC can be misdiagnosed as invasive ductal carcinoma or other basal-like subtypes, making accurate histopathological recognition essential. 

Historically, mastectomy has been favored, especially in multifocal presentations, yet recent evidence supports breast-conserving surgery with adjuvant radiotherapy as an effective and less morbid alternative [4-6]. The rationale for radiotherapy stems from studies demonstrating comparable or improved disease-specific survival in patients receiving adjuvant treatment after lumpectomy [5]. In this report, we present a rare case of multifocal ACC of the breast treated with breast-conserving surgery and adjuvant radiotherapy. This case highlights the diagnostic complexity and therapeutic decision-making in uncommon multifocal presentations, contributing to the limited body of evidence that supports conservative management.

## Case presentation

A 56-year-old woman presented for a second opinion in October 2024 following abnormal mammography and ultrasound imaging findings and a palpable, painless mass in her right breast. Her medical history was otherwise unremarkable, with no relevant personal or familial breast cancer history.

Physical examination revealed two distinct lesions: subtle skin retraction at the 4 o'clock position, approximately 1 cm from the nipple, without a palpable mass underneath, and a small, approximately 1 cm, nodule at the 10 o'clock position, located 7 cm from the nipple. Additionally, a mobile axillary lymph node was palpable, but showed no inflammatory signs.

**Figure 1:**
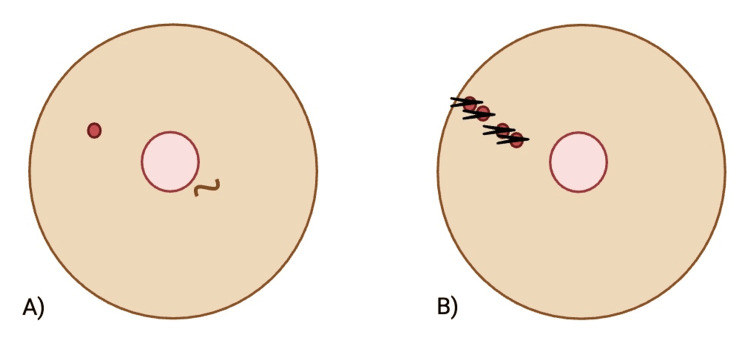
Schematic representation of breast findings (A) Physical exam findings show a nodule at the 10 o'clock position located 7 cm from the nipple, and subtle skin retraction (represented by the curly line) at the 4 o'clock position, approximately 1 cm from the nipple. (B) Breast ultrasound findings exhibit four lesions at the 10 o'clock position located 11 cm, 10 cm, 7 cm, and 6 cm from the nipple. The arrowheads represent the masses that were biopsied. Image Credit: Authors; created in https://BioRender.com. Figures not drawn to scale.

Initial external mammography revealed a 28 × 39 mm dense, irregular nodule with spiculated margins associated with architectural distortion, classified as BI-RADS (Breast Imaging Reporting and Data System)5. Breast density was categorized as type C (heterogeneously dense). Targeted breast ultrasound confirmed multiple lesions at distinct radii: a 16 × 11 × 10 mm irregular solid lesion with angular and spiculated margins at the 10 o'clock position 11 cm from the nipple, a 10 × 7 × 7 mm solid lesion with poorly defined margins at the 10 o'clock position 10 cm from the nipple, a 26 × 16 × 20 mm polylobulated solid lesion at the 10 o'clock position 7 cm from the nipple, and a smaller irregular solid lesion measuring 6 × 5 × 5 mm at the 10 o'clock position 6 cm from the nipple. No mass was observed at or near the 4 o'clock position. Axillary ultrasound demonstrated normal-appearing lymph nodes. A schematic representation of both the physical exam and ultrasound findings is given in Figure 1.

**Figure 2:**
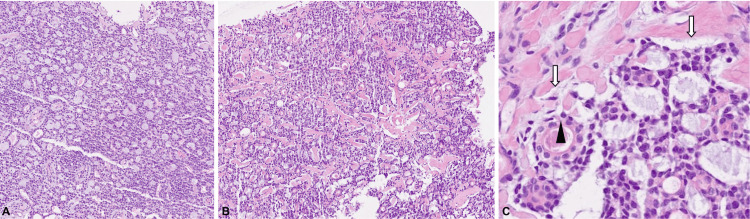
Histological sections of biopsied tumor Histological sections show an infiltrative neoplasm with a predominantly cribriform architecture. Numerous pseudocystic spaces filled with basophilic mucinous material are present. The tumor consists of small nests and cords of epithelial cells arranged in a biphasic pattern, with mild nuclear atypia and low mitotic activity (A) (H&E, 10X). This section also reveals a cribriform pattern with more prominent stromal separation. Some luminal spaces contain dense eosinophilic basement membrane-like material. The neoplastic cells show uniform round nuclei and sparse mitotic figures (B) (H&E, 10X). The neoplasm exhibits a dual cell population (C). The epithelial layer (arrowhead) surrounding the pseudolumens consists of cuboidal cells with dense eosinophilic cytoplasm and oval nuclei without atypia. Additionally, there is a myoepithelial layer (white arrow), slightly flattened, with clear to pale eosinophilic cytoplasm (H&E, 40X).

Ultrasound-guided core needle biopsies were performed on all four lesions located at the 10 o'clock position due to the high suspicion of malignancy. Histopathological examination revealed ACC in all biopsied lesions (Figure 2). These findings included malignant epithelial infiltrating neoplastic lesions of the mammary gland, displaying a cribiform and tubular pattern arranged in anastomosing cords and small nests diffusely infiltrating the breast tissue. These structures appeared to be lined by two distinct cell populations: a peripheral layer of flattened, pale eosinophilic myoepithelial cells, and a luminal layer composed of one or more layers of eosinophilic epithelial cells. The neoplastic cells exhibited mildly irregular, enlarged nuclei with chromatin and small eosinophilic nucleoli. Mitotic activity was very low (Ki-67 of 5%).

**Figure 3:**
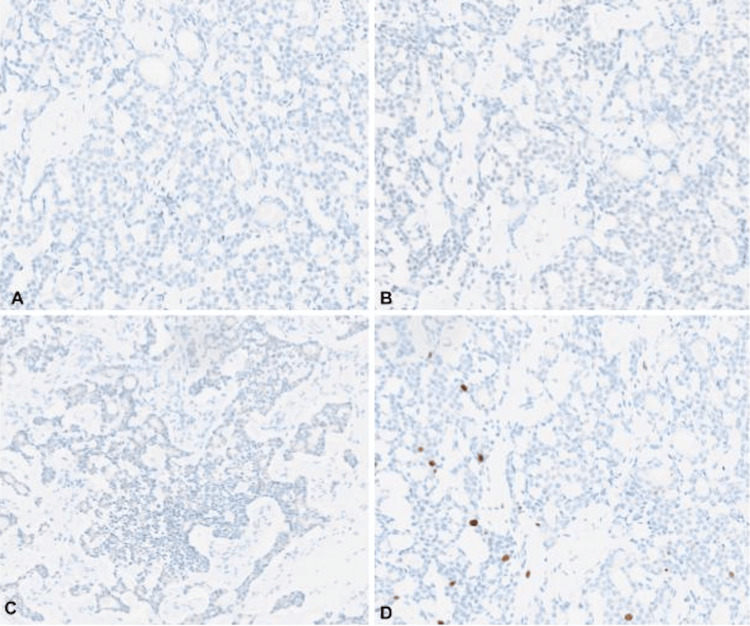
Immunohistochemistry for estrogen, progesterone, and HER2-Neu receptors Immunohistochemistry for estrogen receptors (A), progesterone receptors (B), and HER2-Neu (C) showed negative staining in neoplastic cells, accompanied by a low Ki67 proliferation index of 5% (D) (20X). HER2: human epidermal growth factor receptor 2

**Figure 4:**
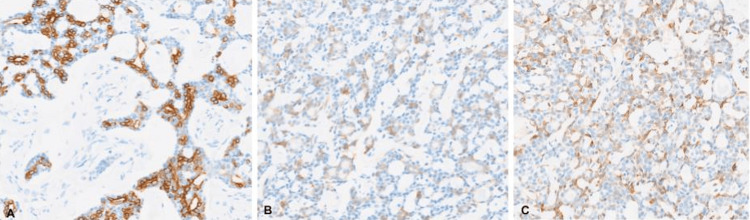
Immunohistochemistry for cytokeratin 7, CD117, and calponin Immunohistochemical staining for cytokeratin 7 (A) and CD117 (C-KIT) (B) highlights the epithelial cell population delineating cystic spaces, whereas calponin (C) staining accentuates the peripheral myoepithelial cell population (200X).

Immunohistochemistry (IHC) demonstrated negativity for ER, PR, and HER2/neu (IHC score of 0) (Figure 3), and positivity for CD117, CK7, and calponin in luminal neoplastic epithelial cells (Figure 4). Ductal carcinoma in situ (DCIS), lobular carcinoma in situ (LCIS), and lymphovascular invasion were not identified in the initial report.

**Figure 5:**
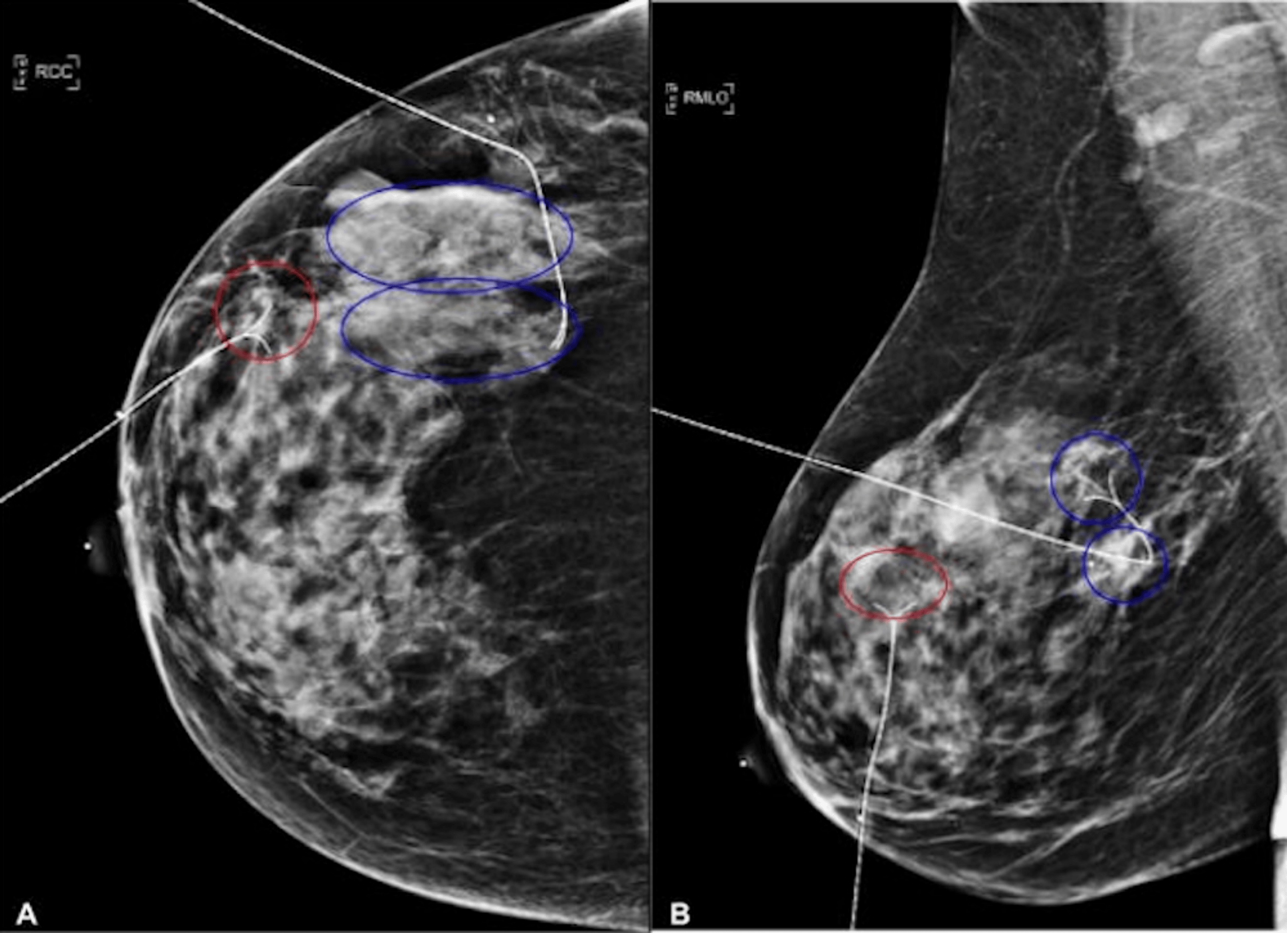
Control mammography post ultrasound-guided wire insertion (A) A right craniocaudal digital mammogram demonstrates two radiopaque localization hook-wires (Bard™ DuaLok™, 20 G × 7.7 cm; Becton, Dickinson and Company, Franklin Lakes, New Jersey, United States) within the breast parenchyma. One wire is directed toward the medial/anterior third of the breast, marking a solitary malignant nodule (encircled in red) in the anterior interquadrant; the second wire is oriented toward the lateral/posterior third, localizing two adjacent malignant nodules (encircled in blue) in the posterior interquadrant. (B) A right mediolateral oblique digital mammogram of the right breast demonstrates two radiopaque localization hook-wires (Bard™ DuaLok™, 20 G × 7.7 cm) traversing the breast parenchyma. One wire is directed toward the anterior third of the breast, marking a solitary malignant nodule (encircled in red) in the anterior interquadrant; the second wire courses into the posterior third, localizing two adjacent malignant nodules (encircled in blue) in the posterior interquadrant. The pectoralis muscle is seen posteriorly.

In February 2025, the patient underwent breast-conserving surgery (partial mastectomy) guided by bracket localization (Figure 5) with adjuvant radiotherapy. No complications occurred during or after surgery. Postoperative evaluation showed excellent wound healing with clean, well-approximated surgical margins and only minor ecchymosis. 

Definitive postoperative pathology confirmed a Nottingham Grade I ACC measuring 2.5 × 2.3 × 1.5 cm. The tumor displayed glandular differentiation (score 2), moderate nuclear pleomorphism (score 2), and a low mitotic index (score 1). DCIS measuring 6 mm, characterized by a solid and cribriform architectural pattern, nuclear grade II without necrosis, was identified. LCIS was absent. Margins were negative for invasive carcinoma, with the closest margin at 1 mm inferiorly. Margins for DCIS were also negative. Additional histological findings included moderate usual-type ductal hyperplasia, tubular and sclerosing adenosis, and fibroadenomatous changes. The final pathological staging was pT2 pN0 M0. A week later, a PET scan was performed, which showed no areas of abnormal fludeoxyglucose (FDG)-uptake, maintaining the TNM status. 

Currently, the patient is undergoing adjuvant radiotherapy without clinical complications and has been recommended for hormonal therapy for five years despite negative hormonal receptor status, a decision warranting further clinical discussion. Adjuvant endocrine therapy was advised due to the coexistent DCIS and areas of lobular intraepithelial neoplasia, which are considered risk factors for recurrence and may benefit from chemoprevention strategies. 

## Discussion

ACC of the breast is an extraordinarily rare tumor, constituting less than 0.1% of all breast cancers and often presenting with nonspecific clinical and imaging features [1,7]. Despite its immunophenotypic overlap with aggressive triple-negative subtypes, ACC characteristically demonstrates a low proliferation index and indolent clinical behavior, as reflected by a Ki-67 labeling of approximately 5% in most reported series [1,2]. In contrast to most published cases presenting with a single tumor focus [1,4], the current patient had four distinct masses, all histologically confirmed, reinforcing the heterogeneity of ACC presentation. This case's multifocal presentation is notably uncommon and serves as a reminder that ACC can manifest beyond the solitary nodules typically described.

Accurate histopathological and immunohistochemical evaluation is critical to distinguishing ACC from other basal-like carcinomas. The absence of estrogen receptor, progesterone receptor, and HER2/neu expression, together with strong CD117 (c-KIT) positivity, confirms the diagnosis of ACC [1,2,4]. Additional markers, CK7 and calponin, highlight the characteristic biphasic epithelial-myoepithelial architecture and facilitate differentiation from benign mimics such as collagenous spherulosis and more aggressive triple-negative carcinomas [3,7]. This precise immunoprofiling prevents unnecessary chemotherapy and extensive surgery that would otherwise be indicated for typical triple-negative cancers. Additionally, *MYB* gene rearrangement or overexpression, identified in the majority of ACCs, may serve as a useful molecular marker, although it is not routinely assessed in clinical practice [1]. 

Surgical management of ACC has traditionally favored mastectomy, especially in multifocal settings. However, recent retrospective analyses support breast-conserving surgery combined with adjuvant radiotherapy as an effective alternative. Sun et al. demonstrated a five-year cause-specific survival rate of 96.1% for patients treated with lumpectomy plus radiotherapy, compared to 91.8% with lumpectomy alone [5], while Gomez-Seoane et al. reported a 10-year disease-specific survival of 94.1% in those receiving postoperative radiation versus 90.2% without [6]. Our patient underwent bracket-guided partial mastectomy with clear margins, followed by radiotherapy, and remains disease-free at four months post-treatment, an outcome consistent with these larger cohorts [8,4]. Although multifocality often leads to more radical surgery, this case exemplifies how accurate diagnosis and careful surgical planning can support a breast-conserving approach even in complex presentations.

No axillary surgical staging was performed in the current case, which aligns with literature suggesting that axillary involvement in ACC is rare and often clinically insignificant [2]. Given the absence of suspicious lymph nodes on physical examination and imaging, and the low risk of nodal metastasis reported in large case series, omission of sentinel lymph node biopsy was considered appropriate and consistent with current evidence-based practice [1,4].

Given ACC's indolent nature and typically low mitotic index, adjuvant chemotherapy is rarely indicated and generally reserved for high-grade tumors [1,2,4], nodal involvement [1,2], or metastatic disease [4], scenarios not present in our patient. In the absence of strong evidence or clear benefit, systemic chemotherapy is usually avoided. Similarly, the decision to recommend endocrine therapy despite hormone receptor negativity remains controversial and lacks consensus in current guidelines. Although some reports suggest potential responsiveness in hormone receptor-positive variants [2], the benefit in triple-negative ACC is unproven. In this case, the recommendation for adjuvant endocrine therapy stemmed from the coexistent low-grade DCIS, measuring 6 mm, and areas of lobular intraepithelial neoplasia identified on the surgical specimen, both of which are considered risk factors for recurrence and may benefit from chemoprevention strategies. The DCIS was completely excised with negative margins. While polemic, such decisions highlight the absence of standardized guidelines and the need for individualized management in rare subtypes. 

While ACC is categorized under the umbrella of triple-negative breast cancers, its prognosis diverges markedly from that of conventional triple-negative ductal carcinomas. Population-based studies demonstrate superior survival and minimal nodal involvement in ACC [4-6], underscoring the need to tailor treatment strategies to its unique biology rather than follow standard triple-negative protocols.

Importantly, although our patient remains disease-free at four months post treatment, this relatively short follow-up period limits conclusions regarding long-term control and highlights the necessity for extended surveillance to ensure durable remission. ACC is known for its indolent course but can exhibit late local or distant recurrence, even beyond 10 years, particularly in cases of high-grade or incompletely excised tumors. This highlights the necessity for prolonged and vigilant surveillance to ensure durable remission. Continued follow-up with clinical exams and imaging is essential, given the potential for delayed recurrence despite initial favorable outcomes. 

## Conclusions

This multifocal ACC case highlights the importance of meticulous immunohistochemical diagnosis to guide conservative surgical management and adjuvant radiotherapy over more radical approaches. Furthermore, multicenter collaborations and the establishment of consensus guidelines are imperative to optimize and standardize care pathways for this rare breast carcinoma subtype.
